# Loci nominally associated with autism from genome-wide analysis show enrichment of brain expression quantitative trait loci but not lymphoblastoid cell line expression quantitative trait loci

**DOI:** 10.1186/2040-2392-3-3

**Published:** 2012-05-16

**Authors:** Lea K Davis, Eric R Gamazon, Emily Kistner-Griffin, Judith A Badner, Chunyu Liu, Edwin H Cook, James S Sutcliffe, Nancy J Cox

**Affiliations:** 1 Section of Genetic Medicine, Department of Medicine, University of Chicago, Chicago, IL 60637, USA; 2 Division of Biostatistics and Epidemiology, Medical University of South Carolina, Charleston, SC, USA; 3 Department of Psychiatry, University of Chicago, Chicago, IL 60637, USA; 4 Department of Psychiatry, University of Illinois, Chicago, IL 60608, USA; 5 Vanderbilt Brain Institute, Vanderbilt University, Nashville, TN 37232, USA

**Keywords:** Autism, annotation, cerebellum, enrichment, expression quantitative trait (eQTL), GWAS, LCL, pannexin, parietal, SLC25A12

## Abstract

**Background:**

Autism spectrum disorder is a severe early onset neurodevelopmental disorder with high heritability but significant heterogeneity. Traditional genome-wide approaches to test for an association of common variants with autism susceptibility risk have met with limited success. However, novel methods to identify moderate risk alleles in attainable sample sizes are now gaining momentum.

**Methods:**

In this study, we utilized publically available genome-wide association study data from the Autism Genome Project and annotated the results (*P* <0.001) for expression quantitative trait loci present in the parietal lobe (GSE35977), cerebellum (GSE35974) and lymphoblastoid cell lines (GSE7761). We then performed a test of enrichment by comparing these results to simulated data conditioned on minor allele frequency to generate an empirical *P*-value indicating statistically significant enrichment of expression quantitative trait loci in top results from the autism genome-wide association study.

**Results:**

Our findings show a global enrichment of brain expression quantitative trait loci, but not lymphoblastoid cell line expression quantitative trait loci, among top single nucleotide polymorphisms from an autism genome-wide association study. Additionally, the data implicates individual genes *SLC25A12*, *PANX1* and *PANX2* as well as pathways previously implicated in autism.

**Conclusions:**

These findings provide supportive rationale for the use of annotation-based approaches to genome-wide association studies.

## Background

Autism spectrum disorder (ASD) is characterized by impairments in three core domains: development of social interaction; language and communication development; and restricted or circumscribed interests and repetitive behaviors [1]. By definition, children with autism show impairments in all three areas, though the degree to which each is affected varies. The study of autism genetics began with twin studies conducted as early as the 1970s [2], indicating a strong heritable component. Since then, the autism genetics field has progressed to include linkage, association, copy number variation and candidate gene studies targeting the broad autism diagnosis as well as specific endophenotypes of autism. The discovery of genetic variants involved in autism has been confounded by phenotypic, genetic and allelic heterogeneity, multiple modes of transmission, inconsistent application of diagnostic criteria and unknown environmental risks. Progress has been made in our understanding of the genetic architecture of autism despite these complexities. However, few genes harboring common risk factors have been convincingly implicated in the etiology of autism.

Multiple traditional genome-wide association studies (GWAS) of ASD have been published with limited power to detect common variants contributing to risk. Genome-wide significantly associated risk alleles have been identified on chromosome 5p14 [3,4], 5p15 [5] and 20p12.1 [6]. The latter study was the largest to date and included over 1,500 parent–child-trio families in the analysis. The single genome-wide significant result found on chromosome 20p12.1 fell within the *MACRO domain containing protein 2* gene. However, at least one subsequent study has failed to replicate this association [7].

Recent studies have shown that novel analytic approaches using GWAS data can increase the power to identify statistically significant relationships. For example, Lu and Cantor describe a method that allows for sex-specific associations, resulting in highly significant findings at the ryanodine receptor 2 locus (*P* <3.9 × 10^-11^) in a multiplex autism dataset [8]. Additionally, several pathway-based analyses of autism GWAS have recently been published, showing enrichment of sub-genome-wide significant associations with pathways of prior interest in autism. For example, Yaspan and colleagues applied their method (pathway analysis by randomization incorporating structure) to the available Autism Genetic Resource Exchange GWAS dataset, subsequently identifying over-representation of pathways involved in ubiquitination (supporting a pathway analysis in Glessner *et al*. [9]) as well as synthesis and degradation of ketone bodies [10]. Anney and colleagues also published a pathway-based analysis, which successfully applied the SNP ratio test methodology to genome-wide data from Autism Genome Project (AGP) families, resulting in a number of convincingly implicated pathways [6]. Mining of existing autism GWAS data is proving to be a valuable undertaking with the potential to provide significant insight into the biology and common risk assessment of ASD.

Recent studies providing thorough investigation of top signals from GWAS of a number of complex disorders have shown enrichment for expression quantitative trait loci (eQTL) in relevant tissues [11-13]. These findings suggest that SNPs with significant functional impact on the expression of genes are more likely to be associated with disease phenotypes [11]. Based on this finding, our group sought to determine if brain-specific eQTL were over-represented in the most significant association from a recently published autism GWAS and whether the specific genes implicated as enriched might highlight new, or help cement existing, candidate genes and pathways. We used publically available data from the AGP GWAS [6], the SNP and CNV Annotation Database [14] and genome-wide expression datasets in brain [13] to test for enrichment of eQTL (those previously identified in transformed lymphocytes and two regions of the brain, parietal lobe and cerebellum), among top nominally associated SNPs from reported autism GWAS.

## Methods

### Study design

**Figure 1:**
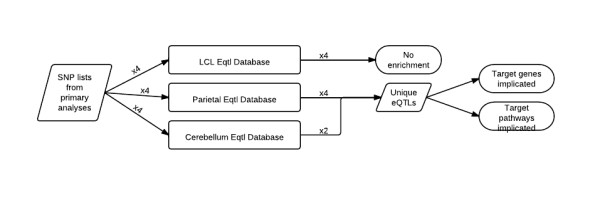
** A flowchart showing the study design.** One SNP list from each of the four primary analyses in the publication from Anney *et al*. [6] (totaling four lists of SNPs) was annotated for eQTL information against an LCL eQTL dataset (http://www.SCANdb.org[14]), parietal tissue and cerebellar tissue. No enrichment was found in any of the LCL annotated datasets, enrichment was found in the parietal lobe for all four analyses and in cerebellar tissue for two of the datasets. The eQTL found in each of the six analyses were combined in order to identify unique eQTL signals that appeared in one or more tissues and in one or more primary analyses. This complete list of unique eQTL signals was then investigated for genes and pathways of interest in autism. eQTL: expression quantitative trait loci; LCL: lymphoblastoid cell line.

This study was designed to detect enrichment of brain eQTL amongst top signals from the recent AGP GWAS by Anney *et al*. [6]. All human subjects research in the study by Anney et al. was conducted in accordance with the Helsinki Declaration and with oversight by the appropriate site-specific Internal Review Board [6]. A schematic of the method utilized in this study is presented in Figure1. Briefly, four total lists of SNPs were collected from the additional files of the paper by Anney *et al*. These include one SNP list from each primary analysis (Spectrum diagnosis [Spec]|All Ancestry [All]; Strict diagnosis [Strict]|All; Spec|Western European Ancestry [WestEur]; Strict|WestEur) meeting a *GWAS P*-value threshold of 10^-3^. Three of these analyses reflect strata of the broadest diagnostic (spectrum) and ancestry (all) categories and therefore these analyses are overlapping and non-independent. The top SNPs from each of these GWAS were analyzed for eQTL enrichment in two different brain tissues (parietal and cerebellum) as well as transformed lymphoblastoid cell lines (LCLs), as described below. Comparison of each SNP list to a randomized null distribution of SNPs (described below) within each eQTL database yielded an empirically determined *enrichment P*-value. Furthermore, the targets for those eQTL present within the four AGP GWAS lists and showing enrichment in brain tissues were identified. Additional analyses were conducted on these eQTL targets to determine if particular genes, gene families or pathways were strongly implicated by eQTL present in any of the primary AGP GWAS analyses.

### Autism genome project genome-wide association study data collection

The methods and samples used for the AGP GWAS are described in depth in the publication by Anney *et al*. [6]. Additional files published with that analysis contained all SNPs significant at *P* <0.001 under an additive model from which the primary analyses were extracted. The lists of significant SNPs came from family-based primary analyses including those stratified by diagnosis subtype and population. In total, four lists of SNPs, significant at a *GWAS P* <10^-3^ threshold, were taken from the publically available GWAS results published by Anney *et al*. [6] (Table1).

**Table 1:** Enrichment results for primary analyses

**Diagnosis**	**Ancestry**	**Parietal enrichment**	**Cerebellar enrichment**	**Lymphoblastoid cell line enrichment**
Spectrum	All	*P* = 0.004	*P* = 0.003	*P* = 0.502
*N*_*snps*_ *= 1262*				
*N*_*subs*_ *= 1385*				
Strict	All	*P* <0.001	*P* = 0.872	*P* = 0.110
*N*_*snps*_ *= 991*				
*N*_*subs*_ *= 812*				
Spectrum	Western European	*P* = 0.005	*P* = 0.012	*P* = 0.503
*N*_*snps*_ *= 1209*				
*N*_*subs*_ *= 1230*				
Strict	Western European	*P* <0.001	*P* = 0.451	*P* = 0.496
*N*_*snps*_ *= 998*				
*N*_*subs*_ *= 720*				

Results for primary analyses are shown by diagnostic category and ancestry. The number of subjects (N_subs_) that went into the GWAS, and the subsequent number of SNPs (N_snps_) that were significant at a *GWAS P* <10^-3^ and went into the eQTL enrichment analysis is shown in each diagnostic and ancestry category. The empirically derived *enrichment P*-value for each diagnostic and ancestry category in each tissue tested is also provided. eQTL: expression quantitative trait loci; GWAS: genome-wide association study; SNP: single nucleotide polymorphism.

### Expression quantitative trait locus determination

eQTL data from LCLs were generated from 176 HapMap European (CEU) and African (YRI) cell lines as described previously (GSE7761) [15]. Cerebellar (GSE35974) and parietal cortex (GSE35977) cis and trans eQTL were generated from 153 individuals of European ancestry obtained from the Stanley Medical Research Institute [13]. ComBat and Surrogate Variable Analysis were used to adjust for batch and both known and unknown covariate effects [16,17]. SNP genotyping was performed on the Affymetrix Genome-Wide Human SNP Array 5.0 (Affymetrix, Santa Clara, CA, USA) and imputed, using MaCH to include all HapMap SNPs [18,19]. The Affymetrix Human Gene 1.0 ST Exon Array was used for gene expression profiling. Imputed genotype dosage data were analyzed for association with expression phenotypes using PLINK [20]. For cis eQTL, defined as SNPs within 4 Mb of the probe site, the corrected significance threshold for determining eQTL status (*eQTL P*-value) was *P* <0.0001. For trans eQTL, the *eQTL P*-value was corrected as 0.05/n, where n = 25,834 probes for the expression data.

### Enrichment of top genome-wide association study signal analysis

All ASD GWAS SNPs with a *GWAS P* ≤10^-3^ in the four primary analyses were subsequently annotated with eQTL information, including the strength of the evidence for the impact of the polymorphism on expression. To test for enrichment of eQTL among these top SNP associations, 1,000 randomized SNP sets were generated. Each of these 1,000 lists, comprising the simulated set of SNPs, included the same number of SNPs as the original list of ASD GWAS associations and contained only SNPs matching the minor allele frequency distribution of the original list of ASD GWAS SNPs. The random lists were sampled without replacement from the set of typed SNPs on the Illumina HumanHap610 array. Minor allele frequency matching was conducted by classifying all Illumina Hap610 platform SNPs into discrete minor allele frequency bins at 5% intervals (0 to 5%, 5 to 10%, …,45 to 50%), followed by random selection of SNPs from the same allele-frequency bins as those in the top signals. The number of eQTL in each simulated set yields an empirical distribution and an *enrichment P*-value, calculated as the proportion of randomized sets in which the eQTL count matches or exceeds the actual observed count in the list of top SNP associations.

### Pathway analysis of significantly enriched brain expression quantitative trait loci

The program PANTHER (http://www.pantherdb.org[21]) was used to identify networks or gene families represented by top autism GWAS eQTL. PANTHER’s classification of genes and proteins provides functionality to determine if gene annotations (that is, membership in pathway or Gene Ontology) are over-represented in a test gene list compared with expectation, given all genes in the human genome. *P*-values generated by the program are Bonferonni-corrected for the number of comparisons conducted. Of the 140 unique genes implicated as eQTL targets from the four primary autism GWAS, 130 were mapped to identifiers in the PANTHER database and analyzed for pathway, molecular function and biological process terms that were over-represented compared to expectation.

## Results

The focus of this study was to determine if enrichment of brain eQTL (that is, SNPs that regulate brain expressed genes) existed in the top signals from published autism GWASs. To this end, we analyzed SNPs at a *GWAS P* <10^-3^ for four primary analyses from the published AGP GWAS [6].

### Primary genome-wide association study enrichment analyses

**Figure 2:**
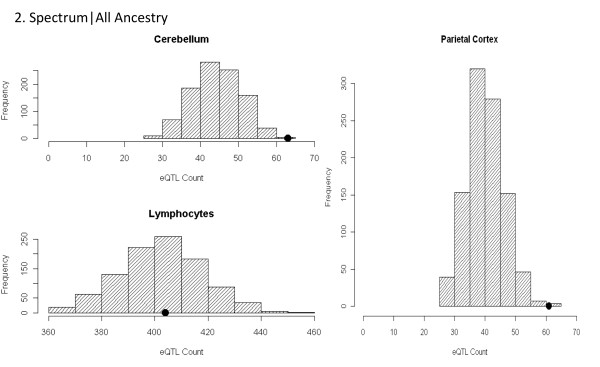
** Histograms of expression quantitative trait loci enrichment analyses for Spect|All analyses.** The gray histogram distributions represent the null distribution created by 1,000 lists of randomly ascertained SNPs drawn from the 1 M array platform and conditioned on minor allele frequency. The black dot represents the actual count of SNPs annotated as eQTL present in each tissue in the Spectrum|All Ancestry analysis. We observed significant enrichment of parietal (*P* <0.004) and cerebellar (*P* <0.003) eQTL, but not LCL eQTL (*P* <0.502). eQTL: expression quantitative trait loci; LCL: lymphoblastoid cell line; SNP: single nucleotide polymorphism.

**Figure 3:**
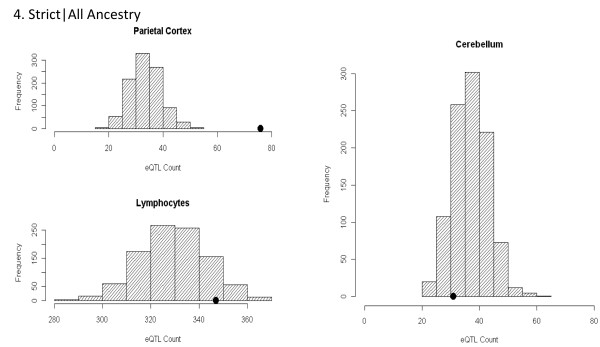
** Histograms of expression quantitative trait loci enrichment analyses for Strict|All dataset.** The gray histogram distributions represent the null distribution created by 1,000 lists of randomly ascertained SNPs drawn from the 1 M array platform and conditioned on minor allele frequency. The black dot represents the actual count of SNPs annotated as eQTL present in each tissue in the Strict|All Ancestry analysis. Significant enrichment of parietal eQTL (*P* <0.001), but not cerebellar (*P* <0.872) or LCL (*P* <0.110) eQTL was identified in the Strict|All dataset. eQTL: expression quantitative trait loci; LCL: lymphoblastoid cell line; SNP: single nucleotide polymorphism.

The primary GWAS enrichment analyses consist of two stratifications including diagnosis and ancestry. We observed significant enrichment of parietal (*P* = 0.004) and cerebellar (*P* <0.003) eQTL, but not LCL eQTL (*P* = 0.502) among the top signals from the most broadly inclusive dataset of spectrum diagnosis including all ancestries (Figure2). Similarly, enrichment of both parietal (*P* = 0.005) and cerebellar (*P* = 0.012) eQTL persisted in the spectrum diagnosis when limited to individuals of Western European descent. Significant enrichment of parietal eQTL (*P* <0.001), but not cerebellar (*P* = 0.872) or LCL eQTL (*P* = 0.110) was identified in the Strict|All dataset (Figure3). We again observed significant enrichment of parietal (*P* <0.001) eQTL but not cerebellar (*P* = 0.45) or LCL (*P* = 0.49) eQTL in the Strict diagnosis when limited to individuals of Western European descent. Results from all analyses are summarized in Table1.

### Descriptive statistics from primary autism genome-wide association study expression quantitative trait loci enrichment analyses

SNPs from all four primary non-independent SNP lists that were eQTL in either cerebellum or parietal tissues contributed to a total number of 539 brain eQTL found in the primary AGP GWAS top signals. In brief, of the total 539 eQTL SNPs, 256 eQTL SNPs were uniquely identified, meaning that the remaining 283 eQTL SNPs were redundancies of the 256 unique eQTL SNPs. Of the unique 256 eQTL SNPs, 214 (84%) were found to be eQTL for only one gene. Forty-two (16%) eQTL SNPs acted as eQTL for more than one gene. Of the 214 SNPs that target only one gene, 124 (58%) act *in cis* and 88 (42%) act *in trans*. Of the 42 SNPs that target more than one gene, 21 (50%) SNPs regulate all target genes *in cis*, 5 (12%) SNPs regulate all target genes *in trans*, and 16 (38%) SNPs are both trans and cis regulators of different target genes. All eQTL from enrichment analysis are provided in Additional file 1: Table S1.

From this total set of SNPs, 140 genes were uniquely implicated as eQTL targets. Of these 140 genes, 62 (44%) were cis implicated and 78 (56%) implicated *in trans*; the proportion of cis implicated genes (in contrast to trans implicated genes) is greater than expected in either cerebellum or parietal tissues based on the observed number of such genes (31% and 28%, respectively). Of the 140 uniquely implicated genes, 71 (51%) genes were implicated in only one analysis; 69 (49%) genes were detected in two or more analyses; 20 (14%) genes were identified in three or more analyses; and 14 (10%) genes were found in all four analyses. There was relatively little overlap in the eQTL target genes across tissues. In an analysis of the broadest diagnostic and ancestry category, only 18 (13%) of the 140 brain-enriched genes were also targeted by eQTL found in LCLs. Of these 18, only three (2%) were identified in parietal, cerebellum and LCL tissues. Of the 140 brain-enriched genes, only 10 genes (7%) were found in both cerebellum and parietal tissues. In all pairwise comparisons of tissues, the overlap in the eQTL target genes was not statistically significant (*P* >0.05, Fisher’s exact test). All genes implicated in enrichment analyses are given in Additional file 2: Table S2.

### Gene and pathway level analysis from primary autism genome-wide association study expression quantitative trait loci enrichment analyses

**Figure 4:**
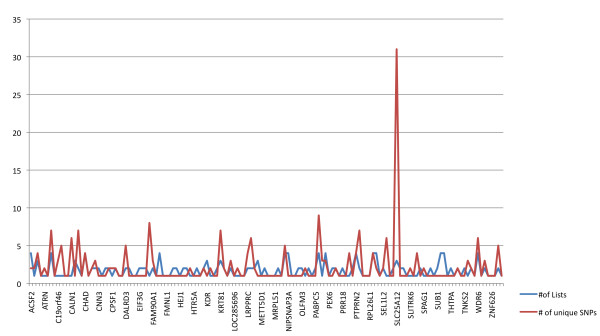
** Bar graph showing the number of expression quantitative trait loci signals across representative loci.** The red line represents the actual number of SNPs implicating each gene. The number of SNPs implicating each gene is not significantly correlated with the gene size (r = 0.13). The blue line represents the number of analyses in which the gene was implicated. The number of analyses in which the gene was implicated is not significantly correlated with gene size (r = 0.09). A subset of genes is listed along the X-axis.

**Figure 5:**
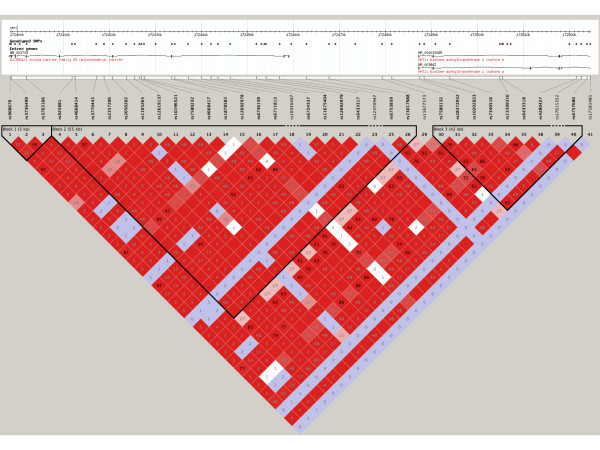
** Linkage disequilibrium structure of*SLC25A12*autism associated expression quantitative trait loci.** Figure was generated using Haploview and demonstrates the linkage disequilibrium structure of only those autism associated eQTL SNPs. Red shading is according to D’ values and r^2^ values are provided in the figure. Three haplotype blocks are identified, extending past the boundary of *SLC25A12*. SNPs in linkage disequilibrium (European, Yorubin and Han Chinese + Japanese) with the SNPs shown here are provided in Additional file 3: Table S3. Four SNPs provide 100% haplotype capture and are also provided in Additional file 3: Table S3. eQTL: expression quantitative trait loci; SNP: single nucleotide polymorphism.

**Figure 6:**
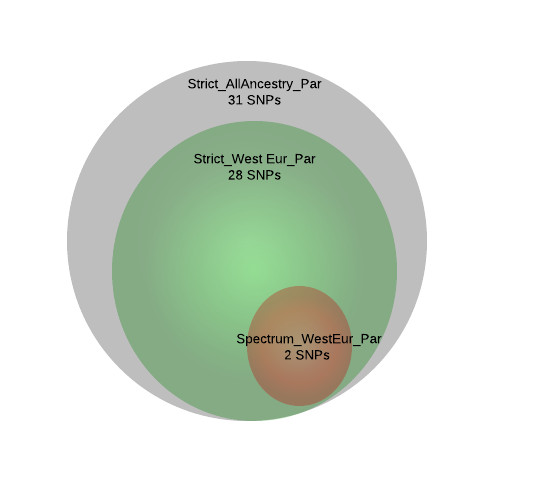
** Venn diagram of the single nucleotide polymorphisms implicating*SLC25A12*.** Thirty-one SNPs were identified in the Strict|AllAncestry analysis as *cis* eQTL regulating *SLC25A12* in parietal tissue. Twenty-eight of those thirty-one SNPs were also identified as cis eQTL for *SLC25A12* in parietal tissue in the Strict|WestEur analysis. Finally, two of those twenty-eight SNPs were also identified as cis eQTL in parietal tissue in the Spect|WestEur analysis. eQTL: expression quantitative trait loci; SNP: single nucleotide polymorphism.

We reviewed the function of those genes implicated in the enrichment analysis to identify common pathways, gene families or functions of said genes. The genes solute carrier family 25 member 12 (*SLC25A12*), pannexin 1 (*PANX1*) and pannexin 2 (*PANX2*) were strongly implicated in our analyses. *SLC25A12* was multiply-implicated by a unique set of 31 SNPs, which was by far the largest number of SNPs implicating a single transcript and was the only transcript implicated in such a way (Figure4). These SNPs all show a high degree of linkage disequilibrium and appear to implicate an extended haplotype (Figure5). Twenty-eight of the 31 SNPs were found in more than one analysis and two of those 28 SNPs were found in three analyses (Figure6). All SNPs implicating *SLC25A12* were cis regulators. All eQTL SNPs implicating *SLC25A12*, and additional information such as SNPs in linkage disequilibrium (r2 > 0.8), are provided in Additional file 3: Table S3.The genes *PANX1* and *PANX2* were also multiply-implicated. *PANX1* was identified as an eQTL target in all four of the primary analyses. No genes were implicated by both cis and trans eQTL; however, *PANX1* was targeted by nine cis eQTL and *PANX2* was targeted by three trans eQTL.

Additionally, we sought to identify any overlap between the eQTL implicated in this study and those recently published by Voineagu and colleagues [22], who identified a number of genes differentially expressed in autism and matched control brains. We found that four of the 140 brain eQTL target genes from our list overlapped with the 1,153 differentially expressed genes identified by Voineagu *et al*. [22]. These included *SLC25A12PANX2calponin 3 acidic* and *cleavage* and *polyadenylation specificity factor subunit 1*.

Based on PANTHER analysis, we found modest evidence of significant over-representation of 14 processes and pathways of interest in autism, including structural components of ribosomes (*P* <0.009), methyltransferase activity (*P* < 0.05), the nicotinic acetylcholine receptor signaling pathway (*P* < 0.02), sensory perception (*P* < 0.01), and receptor activity (*P* < 0.05) among others (Table2).

**Table 2:** Pathway analyses of expression quantitative trait loci implicated genes

	**Analysis type**	***Homo sapiens*genes – REFLIST (19,911)**	**Actual number of autism eQTL genes (140)**	**Expected number of autism eQTL genes**	**Autism eQTL genes (over/under)**	**Autism eQTL genes*P***
Thiamine metabolism	Pathway	2	1	0.01	+	0.01
Nicotinic acetylcholine receptor signaling	Pathway	97	3	0.63	+	0.03
P53 pathway feedback loops 1	Pathway	7	1	0.05	+	0.05
Sensory perception	Biological process	708	10	4.62	+	0.02
Sensory perception of sound	Biological process	123	3	0.8	+	0.05
Carbohydrate metabolic process	Biological process	952	2	6.22	-	0.05
Cell motion	Biological process	964	11	6.29	+	0.05
Cell adhesion	Biological process	1333	14	8.7	+	0.05
Immune system process	Biological process	2628	24	17.16	+	0.05
Cell surface receptor linked signal transduction	Biological process	2235	21	14.59	+	0.05
Structural constituent of ribosome	Molecular function	194	5	1.27	+	0.009
Hydrolase activity	Molecular function	2236	8	14.6	-	0.4
Receptor activity	Molecular function	1808	18	11.8	+	0.5
Methyltransferase activity	Molecular function	132	3	0.86	+	0.05

Analyses were conducted using the program PANTHER. All genes (N = 140) regulated by cis or trans acting eQTL were included in analysis to identify over-representation of pathways, biological processes and molecular functions. A reference list (REFLIST) of annotated genes from the *Homo sapiens* genome (N = 19,911) provided by PANTHER was used as a comparison to the autism eQTL gene list. One hundred and thirty autism eQTL implicated genes were represented in the PANTHER databases, 10 eQTL target genes were not present in the PANTHER accessible databases. The type of pathway, biological process and molecular function showing enrichment among these eQTL targets is provided as is the number of genes annotated to such data in the REFLIST, and in the comparison autism list. Additionally, the number of genes expected in the autism eQTL list, given the number of genes in the REFLIST, is included. The directionality of over- or under-representation in the PANTHER annotation database is included as well as the Bonferroni corrected *P*-value indicating the statistical significance of the over- or under-representation of the biological process, molecular function or pathway in the autism eQTL gene list. eQTL: expression quantitative trait loci.

## Discussion

This study sought to identify biologically meaningful associations buried within top signals that did not meet genome-wide significance thresholds from autism GWAS results. Traditional GWAS in autism have been burdened with problems of heterogeneity and limited sample sizes. To date, multiple traditional autism GWAS have been published although it is important to note that some of these studies use overlapping datasets [3-6]. We believe that, while these studies might be underpowered to identify signals that survive genome-wide correction, their reported results do not reflect the full value of the data.

There are excellent examples to date of such novel methodologies providing additional insight into the role of common genetic variants in complex human disorders, including autism. Anney and colleagues in the AGP also conducted a novel analysis of the present genome-wide data utilizing the SNP ratio test in an effort to detect over-transmitted SNPs in predefined pathways of interest, which subsequently identified multiple pathways and/or Gene Ontology terms enriched for associated signals [23]. Interestingly, two of these pathways, ribosomal components and methyltransferase activity, were also identified in the current study as pathways regulated by eQTL-associated SNPs. Our study complements these pathway-based approaches and demonstrates a method of utilizing expression based SNP annotations to mine already existing analyses for disease associations with functional variants in relevant tissues.

Our study identified significant enrichment of parietal and cerebellum eQTL, but not LCL eQTL, among the top SNP associations in all four of the primary AGP GWAS identified by Anney *et al*. This pattern of results showing significant enrichment in brain (the affected and relevant tissue) and not in tissues peripheral to the main pathology was also seen in a study of cis-regulatory SNPs in bipolar disorder [13]. Similarly, Below *et al*. [12] report enrichment of top signals from a type 2 diabetes GWAS in tissues involved in pathogenesis (muscle and adipose) but not LCLs. Additionally, we found no significant difference in proximity to genes between our GWAS implicated SNPs and the SNPs forming the null distribution. Taken together, the minor allele matching, the lack of a significant difference in distance to nearest gene, and the fact that enrichment is found in some tissues and not in others provide strong evidence against possible sources of systematic bias.

There was very little overlap in the eQTL targets found in parietal, cerebellum and LCLs. Only three target genes, protein NIP-SNAP homologue 3A (*NIPSNAP3A*), TM2 domain containing protein 2 (*TM2D2*) and copine 1 (*CPNE1*), were present in all three tissues in the analysis of the broadest diagnostic and ancestry categories. These findings also strongly suggest that the most appropriate tissue for further functional work is the tissue of pathology. This poses clear challenges for difficult to access tissues, such as neurons. However, there is significant evidence that human induced pluripotent stem cells, once transformed into neurons, provide an invaluable source of neuronal tissue from live patients in which to study cellular phenotypes [24-26]. Our study highlights the importance of furthering the development of methods that allow access to tissues involved in pathology.

While we expected, and observed, overlap in top signals between each primary GWAS, our findings showed an abundance of unique eQTL and implicated a total of 140 target genes. Additionally, three genes, *SLC25A12PANX1* and *PANX2,* were strongly implicated by our results. Two of these genes, *SLC25A12* and *PANX2*, were also identified in a recent study of genes differentially expressed in the brains of individuals with autism compared to control brains [22]. *PANX2* is a protein implicated by trans eQTL that modulates the timing of neuroprogenitor commitment to a neuronal lineage in the hippocampus [27]. It is located 500 kb proximal to SH3 and multiple ankyrin repeat domains protein 3 in the Phelan-McDermid Syndrome 22q13.33 terminal deletion region that has been implicated in ASD [28]. *PANX1* was implicated *in cis* by multiple SNPs in multiple analyses and across multiple tissues and has recently been shown to play a role in N-methyl-D-aspartic acid-mediated epileptiform activity [29]. In murine models, Panx1 and Panx2 are strongly co-expressed early in the developing brain, specifically in the hippocampus [30].

*SLC25A12* is a 109 kb gene found on chromosome 2q31.1 and functions as a calcium-binding mitochondrial protein, integral in the exchange of aspartate for glutamate across the mitochondrial membrane. Several studies have investigated the role of SLC25A12 in the development of autism and *SLC25A12* SNPs have been implicated [31-34]. Due to the platform used in the original AGP GWAS and quality-based filtering of imputed eQTL data, only one of the previously published *SLC25A12* risk variants (rs908670) was included in our study [31]. Rs908670 was identified as an *SLC25A12* eQTL in parietal tissue. Previous work has found *SLC25A12* to be differentially expressed in autism brains compared to controls [22,35]. One such recent study found *SLC25A12* expression decreased in autism brains compared to controls [22]. We found that the risk alleles of the *SLC25A12* eQTL SNPs identified in the AGP GWAS are correlated with decreased expression of *SLC25A12* in the parietal lobe. We add our findings to this growing body of evidence and suggest that common variation in *SLC25A12* auto-regulates expression and may contribute to autism susceptibility.

Our study is limited by the available analyses and the sample sizes used in the original GWAS. It is with some caution that the comparison of results (for example, Strict|All versus Spec|All) must be interpreted. Enrichment analyses, such as those conducted here, include only the top SNP signals and therefore are sensitive to power and sample size differences between the GWAS scans that provide the data to go into an enrichment analysis. It is not possible to make direct comparisons between GWAS enrichment results that differed with respect to sample size, ranging from 720 subjects (Strict|WestEur) to 1,385 subjects (Spec|All). However, within a single sample (for example, Spec|All or Strict|WestEur), comparisons can easily be made between all tissues including cerebellum, parietal and LCLs.

Additional mining of available GWAS data may provide new insight into the biology of autism while allowing the genetics community to leverage data from smaller studies of GWAS. Our study provides evidence for the hypothesis that SNPs below the genome-wide significant threshold (*P =* 10^-8^) are functionally relevant to the development of autism and may yet contribute to risk. Additionally, our results point specifically to *SLC25A12* and *PANX1/2* and to pathways (such as, methyltransferase, ribosomal components) previously implicated in autism.

## Conclusions

Our results demonstrate that loci moderately associated with autism are more likely to be brain-specific eQTL than randomly sampled loci conditioned on minor allele frequency. Moreover, targets for the eQTL identified among top signals from autism GWAS include compelling candidate genes such as *SLC25A12* and the pannexins. These findings suggest that while very few SNPs from a GWAS reach genome-wide significance, it is likely that an abundance of SNPs at sub-significant thresholds are indeed associated with disease risk. This study provides further rationale for annotation-based approaches to GWAS methods.

## Abbreviations

ASD: autism spectrum disorder; AGP: Autism Genome Project; eQTL: expression quantitative trait loci; GWAS: genome-wide association study; LCL: lymphoblastoid cell line; PANX1: pannexin 1; PANX2: pannexin 2; SNP: single nucleotide polymorphism; SLC25A12: solute carrier family 25 member 12.

## Competing interests

The authors declare they have no competing interests.

## Authors’ contributions

LKD conducted analyses and drafted the manuscript. EG conducted analyses and assisted in drafting the manuscript. EKG assisted in drafting the manuscript. JAB and CL conducted analyses yielding brain eQTL data and participated in the drafting of the manuscript. EHC directed analyses and assisted in drafting the manuscript. JS directed analyses of the study and assisted in drafting the manuscript. NJC conceived of the study, directed analyses and helped to draft the manuscript. All authors read and approved of the final manuscript.

## Supplementary Material

Additional file 1 **Table S1. Includes all eQTL from enrichment analysis identified in parietal and cerebellar tissues, from all of the four primary GWAS analyses.** Worksheets provided in the database include information on the eQTL target gene as well as the eQTL host gene. Click here for file

Additional file 2 Table S2. Provides additional information on each gene that was targeted in cerebellum and parietal tissues by an autism-associated eQTL SNP. Click here for file

Additional file 3 Table S3. Provides additional supplementary data regarding SLC25A12 eQTL including the original GWAS p-values and z-scores, parietal tissue eQTL p-values and beta scores and tagged and proxy SNPs for significant eQTL. Click here for file
